# Change the IUCN Protected Area Categories to Reflect Biodiversity Outcomes

**DOI:** 10.1371/journal.pbio.0060066

**Published:** 2008-03-18

**Authors:** Luigi Boitani, Richard M Cowling, Holly T Dublin, Georgina M Mace, Jeff Parrish, Hugh P Possingham, Robert L Pressey, Carlo Rondinini, Kerrie A Wilson

## Abstract

Current classifications of IUCN protected areas are based on management objectives. Fully revising the category system to reflect each category's contributions to biodiversity would greatly enhance their value as effective tools for conserving biodiversity.

In 1872, United States President Ulysses Grant set aside 2.2 million acres of wilderness, primarily for recreational purposes, as the first formally recognized protected area (PA)—Yellowstone National Park. The concept took hold slowly over the next hundred years, and PAs are now recognized as essential to biodiversity conservation [1] and as irreplaceable tools for species and habitat management and recovery. Today, over 100,000 sites (11.5% of the Earth's land surface) are listed in the World Database on Protected Areas [2]. PAs have always been recognized as having broad roles, but it wasn't until the 1990s that the International Union for the Conservation of Nature (IUCN) included the role of conserving biodiversity in its definition: “An area of land and/or sea especially dedicated to the protection and maintenance of biological diversity, and of natural and associated cultural resources, and managed through legal or other effective means” [3].

Despite this recognition of biodiversity's prime significance, when the IUCN established the classification system for PAs (Box 1) in 1994, it categorized them based on their primary management objectives rather than on their biodiversity features (and associated cultural resources). Each category is defined by broad management approaches to be delivered through appropriate techniques for, among other purposes, regulating visitors, educating the public, controlling the utilization of natural resources, and restoring degraded biological communities.

**Box 1. Current IUCN/World Commission on Protected Areas Categories of PAs and Their Main Management Intents**

Ia: Strict Nature Reserve: managed mainly for science

Ib: Wilderness Area: managed mainly for wilderness protection

II: National Park: managed mainly for ecosystem protection and recreation

III: Natural Monument: managed mainly for conservation of specific natural features

IV: Habitat/Species Management Area: managed mainly for conservation through management intervention

V: Protected Landscape/Seascape: managed mainly for landscape/seascape protection and recreation

VI: Managed Resource Protected Area: managed mainly for the sustainable use of natural ecosystems

The category system was established to reduce the confusion that had arisen from the adoption of many different terms to describe different kinds of PAs, to provide international standards for global and regional accounting and comparisons, and to provide a framework for collecting, handling, and disseminating data about PAs [3]. Despite these advances, the PA category system retains a fundamental flaw: because its focus on management intent is unrelated to the basic goal of promoting the persistence of biodiversity, categories do not reflect the role of PAs in biodiversity conservation.

For example, the priority management objectives for category V (“preservation of species and genetic diversity”) and category IV (“maintenance of environmental services”) are aimed at preserving species. However, without explicitly identifying the biodiversity elements or processes to be preserved and the outcomes to be measured—for example, which species or vegetation types are to be conserved, the improvement in viability of an endangered population to be achieved, the trend in community composition proposed, or the nature of ecosystem processes to be maintained—these objectives remain imprecise, the necessary management activities difficult to define, and the outcomes not amenable to quantitative review. Furthermore, under the current IUCN categories of PAs, Yellowstone National Park falls into category II, and presumably its primary management objectives are, among others available in the list for category II, the following: (a) to protect natural and scenic areas for spiritual, scientific, recreational, and tourist purposes; (b) to perpetuate, in as natural a state as possible, representative examples of biotic communities and species; and (c) to manage visitor use for inspirational, educational, and recreational purposes. Under a new category system based on the contribution of a PA to the conservation of biodiversity, Yellowstone National Park would be ranked (among other criteria) according to the objectives of maintaining self-sustaining populations of the endangered grizzly bear and wolf, and maintaining the natural dynamics of the entire guilds of native carnivores and herbivores.

The limitations of the current category system have become ever more apparent as PAs have assumed greater importance in biodiversity conservation and as the system has been applied beyond its original purpose and capabilities. Of particular concern is the extent to which the system is now adopted to provide baseline information in broad, systematic conservation planning assessments. For example, a PA's contributions to biodiversity conservation objectives are judged based on its category, with “higher” categories (e.g., 1–4) considered to be superior for purposes of biodiversity conservation (e.g., [4]), while categories 5 and 6 often constitute the starting point for expanded, landscape conservation systems. The category system is also used as a basis for determining appropriate activities in PAs and for prioritizing additional investment for countries with poor coverage of PAs in certain IUCN categories, in spite of the tenuous link between the effectiveness of biodiversity conservation and the original management categories.

This issue has not gone unrecognized. The IUCN recently issued a report [5] that reviewed the category system and highlighted problems in its implementation, and has begun the process of reviewing the status and efficacy of the current guidelines for application of the PA categories (http://www.iucn.org/themes/wcpa/theme/categories/summit/summit.html). Many welcome this opportunity [6]. IUCN is also considering expanding the original set of purposes to include goals that are directly linked to biodiversity conservation, including using the category system as a tool in bioregional planning, as well as in large-scale conservation and development planning [5].

Fully revising the category system to reflect each category's contributions to biodiversity would add enormously to the value of PAs as effective conservation tools. Such a redesign would reduce the subjectivity of current classifications in favor of more objective criteria, appropriately based upon definable biological components. The categories should be based around conservation objectives concerning the species, communities, or processes that are to be maintained or restored—including, for example, viability of populations or set of habitat types to be maintained—so that progress and successes can then be monitored and recorded. Toward that end, PAs should be defined using criteria that include any measurable aspects of the particular biodiversity features that are the primary reason for protecting that area in the first place (Box 2). For example, if PAs are established to protect a species or a habitat type, or to contribute to maintaining an ecosystem service such as the provision of clean water, these types of objectives should be the basis for their categorization. Then management actions—such as actively supporting populations, establishing wildlife corridors, and controlling human impacts—would be tailored to reduce threats that compromise those conservation objectives.

**Box 2. Suggestions for Switching from Management to Conservation Outcomes**

Biodiversity includes the diversity and abundance of genes, species, communities, ecosystems, and landscapes, and the ecological processes that link all elements in a dynamic state and deliver ecosystem functions and services. Species and habitat types stand out as the most frequent elements of biodiversity targeted for conservation action [7], and are generally much easier to measure, evaluate, and manage than ecological processes (although there are improving and increasingly measurable approaches to planning for ecological processes [8]). We argue that for the practical purpose of implementing a new system of PA categories, outcomes for species and habitat types should be the primary objectives. Outcomes could be measured explicitly through a number of attributes that have been associated with species and ecosystems, such as phylogenetic uniqueness, vulnerability, irreplaceability, richness, and ecological integrity, which could not only give substance and precision to the PA category objectives but also allow their effectiveness to be monitored.

We recommend modifying and strengthening the current category system in the following ways:

**(a) Relate the categories to defined outcomes for conservation elements.** Not only is the current PA category system based on management objectives rather than conservation objectives, but these objectives are simply statements of intent, not actual management regimes. We suggest that the PA categories be based on the quality and quantity of the contribution of each PA to conservation of biodiversity (and associated sociocultural values), specifying explicitly the attributes that are to be conserved, how they will be maintained over time, and how success will be measured at the level of an individual PA (e.g., richness, integrity) and in the broader context of landscapes (e.g., complementarity, irreplaceability).

The goals and objectives of a PA should be stated in terms of explicit and measurable outcomes that reflect the ecological and related social attributes to be conserved (e.g., the restoration and/or maintenance of a viable population of a critically endangered species, or the maintenance of the condition of the largest remnant of a particular habitat type). This adjustment will allow the contributions of PAs to real outcomes to be monitored and more effectively evaluated [9]. This will also improve their performance as indicators of the effectiveness of management in PAs, and as measures for use in prioritization analyses.

**(b) Quantify advanced biodiversity conservation targets of the categories.** A revised PA category system should build on the basic concept of species or habitat type representation to include measurable outcomes like species persistence, habitat condition, and ecosystem integrity [10]. Methods have been developed to assess the conservation status of species (i.e., IUCN/Species Survival Commission Red List Criteria [11]) that could be applied to evaluate the consistency of a PA's management plan with the objectives of maintaining, restoring, and contributing to improving the status of those species [12]. Building upon the Red List experience and its wide participatory process that involved hundreds of scientists and managers, a set of criteria needs to be developed and approved that is linked to measurable attributes of the biodiversity features that are the primary reason for establishing that PA. This further reinforces the need to consider PAs as networks rather than as individual PAs in isolation—the whole is more than the sum of the parts. Moreover, if the effectiveness of a PA is defined by the conservation objectives that it can achieve, including the threats it can mitigate, then its IUCN category does not depend only on the management inside the PA, but also on the context in which it exists. This means, in turn, that management of activities within zones of influence of PAs also needs to be addressed.

**(c) Link PA categories to conservation planning frameworks and to the monitoring and evaluation of PA management effectiveness**. The pressure-state-response model is an ideal framework upon which to base new IUCN categories: identify relevant threats or pressures facing an individual PA or network of PAs in order to determine the conservation measures or management actions that should be taken to achieve their conservation objectives. Moreover, reduction in threats can act as a measure of conservation outcomes [13].

Systematic conservation planning has become the modus operandi of planning and designing PA systems, and a classification system for PAs that was explicit about conservation objectives for individual PAs or networks would vastly improve the information base for conservation assessment and planning. For example, questions such as the following could be addressed routinely:

1. What is a system of PAs expected to achieve in terms of conservation?
2. What type of PA (or network of PAs) is required to protect specific biodiversity features, including necessary management actions?
3. What attributes should be monitored to evaluate the effectiveness of a PA, given its objectives and its ability to counteract threats to achieve these objectives

The revision of the IUCN category system will be a key topic at the 4th World Conservation Congress (October 2008) and at several other meetings on PAs organized in 2008 under the umbrella of the Convention on Biological Diversity. Therefore, the opportunity now presents itself to initiate the process of shifting the focus of the categories from management intent to conservation outcomes, thus making them fully operational tools in biodiversity conservation.

## Wider Considerations

Developing a new system for PA categories will not be an easy task. PAs are not simply technical tools for conservation—they are value-laden institutions that are deeply rooted in national policies and international agreements. Opening the discussion on a well-established system that is universally adopted is a serious decision, not to be taken lightly. In addition to the benefits we have listed, there will also be costs. Changing the current system might prove costly and painful not only for IUCN, but also for the international conservation community. The transition from one system to another will require dedicated efforts. A change of the category system might prove particularly challenging for developing countries that lack the technical and economic resources to apply techniques of conservation planning, enforce goals related to conservation outcomes, and implement biodiversity monitoring programs. Developed countries and international organizations must be prepared to support the transition with skills and resources where necessary.

Without doubt, the current system is easier to apply than the alternative we propose, and might, therefore, be strongly defended by countries that find it easy to apply and have invested deeply in it. However, IUCN has the right and responsibility to manage the PA category system and, if necessary, to change it. IUCN has a track record for applying the best scientific theory and practice to conservation issues. We urge IUCN to consider the benefits of an extensive review of the PA categories based on the best current approaches to ecology and systematic conservation planning. We recommend that IUCN initiate the process (through an appropriate resolution of the forthcoming World Conservation Congress in 2008) by empowering a group of technical and institutional experts to draft a preliminary set of categories, describing their rationale and likely advantages, for consideration and refinement by the IUCN constituency and beyond.

## Abbreviations

IUCN: International Union for the Conservation of Nature
PA: protected area

## References

[pbio-0060066-b001] Bruner AG, Gullison RE, Rice RE, Fonseca GAB (2001). Effectiveness of parks in protecting tropical biodiversity. Science.

[pbio-0060066-b002] World Commission on Protected Areas (2006). World database on protected areas. http://www.unep-wcmc.org/wdpa/.

[pbio-0060066-b003] IUCN World Commission on Protected Areas, World Conservation Monitoring Centre (1994). Guidelines for protected area management categories. http://www.unep-wcmc.org/protected_areas/categories/eng/index.html.

[pbio-0060066-b004] Rodrigues ASL, Andelmann SJ, Bakarr M, Boitani L, Brooks TM (2004). Effectiveness of the global protected area network in representing species diversity. Nature.

[pbio-0060066-b005] Bishop K, Dudley N, Phillips A, Stolton S (2004). Speaking a common language: The uses and performance of the IUCN System of Management Categories for Protected Areas.

[pbio-0060066-b006] Locke H, Dearden P (2005). Rethinking protected area categories and the new paradigm. Envir Conserv.

[pbio-0060066-b007] Redford KA, Coppolillo P, Sanderson EW, Fonseca GAB, Dinerstein E (2003). Mapping the conservation landscape. Conserv Biol.

[pbio-0060066-b008] Pressey RL, Cabeza M, Watts ME, Cowling RM, Wilson KA (2007). Conservation planning in a changing world. Trends Ecol Evol.

[pbio-0060066-b009] Hockings M (2003). Systems for assessing the effectiveness of management in protected areas. Bioscience.

[pbio-0060066-b010] Gaston KJ, Pressey RL, Margules CR (2002). Persistence and vulnerability: Retaining biodiversity in the landscape and in protected areas. J Biosciences.

[pbio-0060066-b011] Lamoreux J, Akcakaya HR, Bennun L, Collar NJ, Boitani L (2003). Value of the IUCN Red List. Trends Ecol Evol.

[pbio-0060066-b012] Parrish JD, Braun DP, Unnash RS (2003). Are we conserving what we say we are? Measuring ecological integrity within protected areas. Bioscience.

[pbio-0060066-b013] Mace GM, Baillie JEM (2008). The 2010 biodiversity indicators: Challenges for science and policy. Conserv Biol.

